# Signaling Pathways of AXL Receptor Tyrosine Kinase Contribute to the Pathogenetic Mechanisms of Glioblastoma

**DOI:** 10.3390/cells13040361

**Published:** 2024-02-19

**Authors:** Alberto Repici, Alessio Ardizzone, Fabiola De Luca, Lorenzo Colarossi, Angela Prestifilippo, Gabriele Pizzino, Irene Paterniti, Emanuela Esposito, Anna Paola Capra

**Affiliations:** 1 Department of Chemical, Biological, Pharmaceutical and Environmental Sciences, University of Messina, Viale Ferdinando Stagno D’Alcontres, 31, 98166 Messina, Italy; alberto.repici@unime.it (A.R.); aleardizzone@unime.it (A.A.); fabiola.deluca@studenti.unime.it (F.D.L.); ipaterniti@unime.it (I.P.); annapaola.capra@unime.it (A.P.C.); 2 Istituto Oncologico del Mediterraneo, Via Penninazzo 7, 95029 Viagrande, Italy; lorenzo.colarossi@grupposamed.com (L.C.); angela.prestifilippo@grupposamed.com (A.P.); gabriele.pizzino@grupposamed.com (G.P.)

**Keywords:** neuro-oncology, Central Nervous System (CNS), glioblastoma, AXL receptor, neuroinflammation

## Abstract

Brain tumors are a diverse collection of neoplasms affecting the brain with a high prevalence rate in people of all ages around the globe. In this pathological context, glioblastoma, a form of glioma that belongs to the IV-grade astrocytoma group, is the most common and most aggressive form of the primary brain tumors. Indeed, despite the best treatments available including surgery, radiotherapy or a pharmacological approach with Temozolomide, glioblastoma patients’ mortality is still high, within a few months of diagnosis. Therefore, to increase the chances of these patients surviving, it is critical to keep finding novel treatment opportunities. In the past, efforts to treat glioblastoma have mostly concentrated on customized treatment plans that target specific mutations such as epidermal growth factor receptor (EGFR) mutations, Neurotrophic Tyrosine Receptor Kinase (NTRK) fusions, or multiple receptors using multi-kinase inhibitors like Sunitinib and Regorafenib, with varying degrees of success. Here, we focused on the receptor tyrosine kinase AXL that has been identified as a mediator for tumor progression and therapy resistance in various cancer types, including squamous cell tumors, small cell lung cancer, and breast cancer. Activated AXL leads to a significant increase in tumor proliferation, tumor cell migration, and angiogenesis in different in vitro and in vivo models of cancer since this receptor regulates interplay with apoptotic, angiogenic and inflammatory pathways. Based on these premises, in this review we mainly focused on the role of AXL in the course of glioblastoma, considering its primary biological mechanisms and as a possible target for the application of the most recent treatments.

## 1. Introduction

Glioblastoma, a very aggressive and malignant kind of brain cancer, rises from glial cells, cellular components that provide support and maintain the homeostasis of the Central Nervous System (CNS) [1]. The two main clinical forms of glioblastoma are typically described as primary or secondary. Primary glioblastoma is the most common form, accounting for about 95% of cases, and usually develops de novo within 3–6 months in subjects over the age 65 with a peak incidence between 75 and 84 years. Secondary glioblastoma develops from low-grade astrocytomas that have occurred over the course of 10–15 years in young adults aged 20 to 40 years [2]. Many glioblastoma tumors arise in the cerebellum and spinal cord, but most of them are found in the frontal lobes as well as in the temporal and parietal lobes [3].

As indicated by the last epidemiological reports in 2020 and then in 2022, the worldwide incidence of glioblastoma is estimated to be around 4–5 cases per 100,000 people per year, with a major prevalence in older adults, and an increasing age-related risk [4,5], in addition to genetic, environmental, and other risk factors such as exposure to therapeutic ionizing radiation, pesticides, and smoking [6].

Early identification is essential for successful treatments since symptoms of glioblastomas frequently include headaches, seizures, and neurological impairments [7].

Generally, glioblastoma can be diagnosed either by neurological examination or by imaging such as positron emission tomography (PET), computed tomography (CT) or magnetic resonance imaging (MRI) [8]. Sometimes, a differential diagnosis is required, which may include the collection and analysis of tissue samples by biopsy, this is crucial to determine cell types and their level of aggressiveness [9]. In certain cases, genetic tests are also carried out to highlight the possible presence of genetic mutations and/or molecular biomarkers predisposing to the onset of cancer [9].

However, due to its intricate etiopathology and capacity to invade surrounding brain tissue, glioblastoma continues to pose a serious threat to oncology.

Surgery, radiation therapy, and chemotherapy are often used as a multimodal strategy to improve glioblastoma patients’ condition; nevertheless, the prognosis is still poor [10], with a median survival of 12 to 15 months [11]. This highlights the critical need for ongoing research and novel therapeutic approaches to improve outcomes for patients suffering from this debilitating disease. Therefore, to make significant progress toward more potent target therapy, a thorough investigation of other receptor systems may aid in understanding glioblastoma’s complex biological network to successfully fight this huge clinical challenge [12]. In this regard, the expression of the receptor tyrosine kinase AXL was correlated with poor survival in a wide range of malignancies, and it is becoming clear that this receptor plays a significant role in tumor growth and spread [13].

Along with TYRO3 and MER, AXL belongs to the TAM family of receptor tyrosine kinases and the name derived from “anexelekto”, a Greek word. In particular, these receptor tyrosine kinases have three structural features in common: an intracellular domain that contains a catalytically competent kinase identified by a distinct KWIAIES conserved sequence; a single-pass transmembrane domain; and an extracellular domain made up of tandem repeats of immunoglobulin-like and fibronectin type 3 (FN-III)-like [14]. TAM receptor tyrosine kinases are expressed in cancer cells, where they support invasion and survival as well as resistance to different treatments, in addition to their role as important regulators of immune cell activation [13]. Indeed, it is currently recognized that AXL expression is associated with a higher risk of metastasis and an unfavorable prognosis of several solid cancers, such as breast cancer, non-small cell lung carcinoma, ovarian cancer, and clear cell renal carcinoma [14,15,16,17,18,19,20].

Dysregulation of AXL was also found in tumor cells, fibroblasts, vascular cells, and various immune cells [15]. Therefore, in a cancer pathological setting, AXL expression may largely favor the disease’s progression, and as a consequence, AXL inhibition could constitute a potential likely therapeutic target [15]. AXL’s biological role has been probed using a variety of strategies for cancer therapy, such as monoclonal antibodies or small chemical inhibitors that compete with ATP-binding enzymes [21,22,23].

The AXL inhibitor R428 (also called BGB324 or Bemcentinib), which is very selective for AXL among TAMs and other receptor tyrosine kinases, is the subject of several active clinical trials [23]. The administration of Bemcentinib was linked to changes in proteins involved in the metabolism of reactive oxygen species, protein kinase B signaling, and other processes, indicating a significant anti-tumor effect in patients with non-small cell lung cancer [24]. Growth arrest-specific gene 6 (Gas6) is a cytokine that binds to Tyro3, AXL, and Mer receptor tyrosine kinases [25]. Several studies have revealed that Gas6, which is produced by macrophages, interacts with AXL to cause cancer to develop, and AXL has been linked to worse clinical outcomes [25].

Thereby additional strategies to block the GAS6/AXL axis have also been proposed, such as vitamin K antagonists that would lessen GAS6’s capacity to activate AXL and decoy receptors that trap GAS6 [26,27,28]. In addition, in a novel strategy, AXL was used as a cancer antigen for chimeric antigen receptor (CAR)-T cell treatment more recently. For instance, engineered T cells expressing AXL-CAR-T were able to trigger cytokine release and antigen-specific cytotoxicity in triple-negative breast cancer (TNBC), where AXL is overexpressed [29,30].

Thus, considering all the above, this review aims to evaluate the molecular mechanisms behind AXL receptors, with a focus on the therapeutic potential of targeted therapies for the treatment of glioblastoma.

## 2. Glioblastoma: Insights and Biological Characteristics of the Most Aggressive CNS Tumor

Glioblastoma is the most common and severe form of primary brain cancer [31,32] and it is characterized by several features such as a high growth rate, high invasiveness of the surrounding tissues and a wide genetic variability that makes the treatment even more complicated [33].

This complex form of malignancy accounts for about 14.5% of all CNS tumors and 48.6% of malignant CNS tumors [34].

The multitude of features that glioblastoma possesses also negatively affects the whole set of symptoms that patients express, which usually can be headaches and seizures, nausea, vomiting, and general weakness [35] and the symptomatology largely depends on the location of glioblastoma in the brain. Unfortunately, about 70% of patients with glioblastoma, even after treatment, may experience relapses over time or even develop resistance to the treatment regimen, resulting in a low survival rate over 5 years [36].

Risk factors associated with the occurrence of glioblastoma are not yet fully understood; exposure to ionizing radiation, such as those used in radiation therapy or in different forms of environmental exposure may increase the risk of developing glioblastoma [37]. Moreover, it has also been observed that the incidence of glioblastoma increases in some rare genetic conditions, such as neurofibromatosis type 1 or Li-Fraumeni syndrome, which may increase the risk of developing brain tumors, including glioblastoma [38].

According to the Cancer Genome Atlas [39] the complexity of glioblastoma is due to a series of genetic mutations that greatly affect its nature and differ from patient to patient. The most common mutations that often affect the course of the disease include four significant signaling pathways: the pathway of the tumor protein p53 (p53), the signaling pathway of the receptor tyrosine 3-kinase (RTK), the protein Ras and the 3-phosphoinositide kinase (PI3K) pathway [40]. Additionally, in the intricate glioblastoma genomic pattern, it is also quite common to see overexpression of epidermal growth factor receptor (EGFR), mutations of isocitrate dehydrogenase 1 (IDH1) and phosphate and tensin homolog (PTEN) [41], which when altered largely contribute to uncontrolled growth, invasiveness and the ability to escape different checkpoints characteristic of glioblastoma.

Genetic alterations of glioblastoma also affect the ability to escape the normal controls of the innate immune system and also develop resistance to drugs that, of course, adversely affect the efficiency of therapy [42].

The therapeutic regimen required for glioblastoma is often complex, and a multidisciplinary approach is needed, standard therapy may include surgery, radiation therapy, and chemotherapy. However, it is important to note that the therapeutic approach is different for each patient, based on parameters such as the location and size of the glioblastoma or factors such as age and the general state of health [43].

The first step to remove a glioblastoma is through surgery, but the extraordinarily invasive nature of glioblastoma prevents the achievement of the desired result without damaging vital areas of the brain [44]. Other forms of therapy involve radiotherapy since X-rays can eliminate most cancer cells [45], however, the best therapeutic results are achieved by combining radiotherapy followed by chemotherapy.

Drugs most often used are alkylating agents that bind to the DNA of cancer cells preventing them from replicating [46]. The most popular and first-line systemic therapy for glioblastoma is Temozolomide (TMZ), an oral DNA alkylating medication that crosses the blood-brain barrier (BBB), nevertheless, despite the available therapies, the mortality rate of patients affected by glioblastoma remains quite high [47].

Over the years, research has been very focused on understanding this type of cancer, thus stimulating a series of increasingly innovative therapeutic approaches.

The most innovative and encouraging therapies exploit the mechanisms of the immune system to enhance its action against glioblastoma [48]. Thus, immune checkpoint inhibitors capable of suppressing the uncontrolled activity of replicating cancer cells have been studied. Some of these targets are programmed death receptors/ligands (PD/PD-L1) and Cytotoxic T-Lymphocyte Antigen 4/Cluster of differentiation 80 (CTL-4/CD-80) [49]. CAR-T are artificial receptors that have the ability to reroute T lymphocyte immune responses to a particular target antigen. As a result, T cells can provide both immediate and long-term effects by inducing intricate antitumor responses. These new therapies could be a valid way to counteract glioblastoma [50].

Until now, the targets tested by CAR-T cell therapy are the human epidermal growth factor receptor 2 (HER2), variant epidermal growth factor receptor III (EGFRvIII) and alpha receptor 2 of Interleukin-13 (IL-13 Rα2) [51].

In the past, it was common to imagine the brain as an ‘immunological sanctuary’, an organ in which the immune system had limited access or activity and immediate immune response to avoid damage to healthy brain tissue [52]. The consideration was mainly due to two characteristics: the presence of the BBB and the absence of a lymphatic drainage system in the brain [53]. The BBB guarantees total isolation of the brain from the external environment, protecting it from physical and mechanical insults, but thanks to its composition it preserves this delicate organ from viruses, toxic molecules, and other potentially harmful agents [54].

Recent studies have shown the existence of a lymphatic system of the meninges which drains all the cerebrospinal fluid (CSF), macromolecules, and immune cells into the deep cervical lymph nodes [55]. These characteristics affect the environmental conditions of the brain and then affect the development of neoplasms. In fact, recent studies have investigated how and how much the microenvironment of the glioblastoma can interact with the immune system and in some cases exercise an immunosuppressive power [56]. It is widely recognized that glioblastoma can escape the surveillance of the immune system through different mechanisms of release of immunosuppressive factors.

The most studied immunosuppressive markers are Interleukin-10 (IL-10), prostaglandin E2 (PGE-2), and transformation growth factor β (TGF-β) [57]. IL-10 increased secretion in the glioblastoma microenvironment has the effect of amplifying the release of other anti-inflammatory cytokines such as IL-4 and Chemokine C-C motif ligand 2 (CCL2) [58]. Besides, IL-10 affects the activity of macrophages presenting the antigen by altering the activation of CD4^+^ T lymphocytes [59].

Also, the release of TGF-β causes CD4^+^ T cells to upturn Foxp3 levels, and then differentiate into T-reg cells with strong immunosuppressive activity [60]. The immunosuppressive activity of PGE-2 is linked to an incorrect function of the expansion of myeloid-derived suppressor cells (MDSCs) [61]. These considerations confirm the complexity and severity of glioblastoma, highlighting how it is still an unresolved clinical challenge.

## 3. Characteristics and Functions of AXL Receptor Tyrosine Kinase

In the general understanding of glioblastoma, it becomes imperative to figure out more suitable therapeutic approaches, and in this pathological context, the interactions with AXL are becoming widely more studied.

AXL is a protein that belongs to the extended family of TAM (TYRO3, AXL and MERTK), a series of receptors that are part of the receptor tyrosine kinases (RTKs) family [62].

The structure of AXL is formed by three parts, one protrudes towards the extracellular matrix, one is transmembrane, and one is intracellular [63]. The gene encoding the protein AXL is found on chromosome 19, precisely 19q13.1 [64], and was first isolated from the primary cells of human myeloid leukemia [65]. The extracellular part is formed in turn by several subunits: two immunoglobulin-like (Ig) and fibronectin type-III (FN III)-like domains which together bind the ligand of this receptor [66]. The ligand that allows the activation of AXL is the GAS6, encoded by the *GAS6* gene [67]. Following activation by GAS6, the intracellular domain of AXL undergoes an auto-phosphorylation that leads to activation of kinase activity [68]. GAS6 is a vitamin-K-dependent growth factor expressed by several cells like monocytes, endothelial cells, brain, heart, fibroblast, and tumor cells [69,70] and has the best affinity for AXL among all the TAM family members.

Canonical activation of AXL occurs via GAS6, but other small molecules capable of binding to Axl are protein S, Tubby, Tubby-like protein 1 (TULP-1) and Galectin-3 [71]. Although GAS6 can bind to other receptors in the TAM family, AXL affinity is about 3–10 times higher [71].

**Figure 1:**
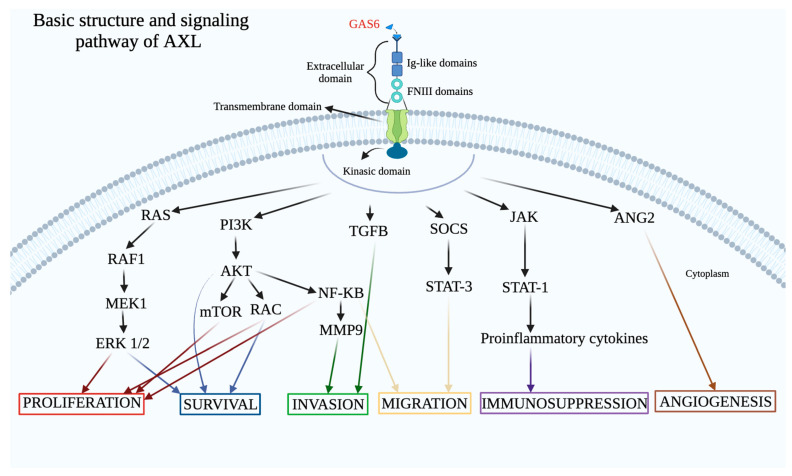
The basic structure and signaling pathway of AXL.

AXL activation leads to the transfer of the signal from the extracellular matrix into the cytoplasm with the consequent triggering of several pathways like PI3K/AKT/mTOR, MAPK and JAK/STAT [72] as summarized in Figure 1.

Recurrent or aberrant activation of AXL has been observed in several pathologies that exploit to their advantage the activation of pathways linked to AXL, in particular, the diseases that most exploit this ‘jammed’ mechanism are cancer, chronic immune disorders and cardiovascular disease [14].

The interplay between the GAS6/AXL axis plays a fundamental role not only in the development of cancer but also in the physiological processes of cell differentiation and proliferation, survival, aggregation, and inflammation [73,74]. It should be noted that the activation of the ligand-dependent AXL pathway is achieved through self-transphosphorylation of tyrosine residues that are present in the kinase domain at the intracellular level [73,74]. Complete activation of AXL occurs only when it interacts with the phospholipid phosphatidyl serine, this process is made possible by the gamma-carboxyglutamic acid (Gla) domain of GAS6 after post-translational modification [73,74]. Phosphatidyl serine is a phospholipid that is typically found in the intracellular region of the phospholipid bilayer; nevertheless, it can become externalized in stressed or dying cells, including those that have been infected by viruses. Because of the elevated apoptotic index of tumors, metabolically challenged tumor cells, vasculature inside the tumor, and exosomes produced from the tumor, the tumor microenvironment also has a high quantity of externalized phosphatidyl serine [75].

The activation of AXL through its ligand Gas6 has as repercussions a wide range of negative and positive actions. Clearly, the physiological responses to the activation of this receptor are altered in the case of glioblastoma. There are several factors not entirely deepened by recent research that are closely related to the downstream action of activated AXL, such as iNOS/NO. In contexts other than glioblastoma Rigoni et al. [76] evaluated the action between iNOS and AXL in relation to the immune response during Trypanosoma cruzi infection. The results showed that Axl-deficient macrophages showed improved effector response, with a high expression of iNOS, which is a marker of M1 responses. This suggests that AXL could suppress the induction of M1 effector macrophages. In addition, it was observed that AXL-deficient macrophages showed better control of parasitic infection. Therefore, the correlation between iNOS and AXL may affect the immune response during Trypanosoma cruzi infection, with suppressive activity on the M1 response and a better control of parasitic infection [76]. Another study on pancreatic cancer analyzed the correlation between the TAM receptor family (TYRO3, AXL, MERTK) [77]. It was found that in KIC (Kras*^LSL-G12D/+^*, Ink4a/Ar^f*lox/lox*^, Ptf1a*^Cre/+^*) tumor microenvironment is strongly chemoresistant. The microenvironment of KIC tumors lacking AXL is more inflammatory, with increased infiltration of T and NK cells and a significant drop in tumor-associated macrophages. These macrophages also have polarized from an immunosuppressive (Arginase-1+) to an anti-tumor (iNOS+) phenotype. This is in line with AXL’s role in efferocytosis, where it has been demonstrated to stimulate macrophage phagocytosis of apoptotic cells [77].

Thus, considering the influence of AXL on iNOS/NO expression and the biological implications for tumor environment caused by this crosstalk, more evidence of the link between AXL and the pro-inflammatory role of iNOS will certainly emerge in the future, especially in glioblastoma.

Furthermore, the activation of downstream pathways and the phosphorylation sites on AXL are very context-dependent and the intracellular domain of AXL contains many tyrosine phosphorylation sites, including Y698, Y702, Y703, Y779, and Y821 [78].

Alterations of the mechanism of AXL (but also of other RTKs) have led to the consideration of such receptors as proto-oncogenes. The forms of hyperactivity linked to AXL are involved in the manifestation of hallmarks of some types of cancer [79]. After being activated, AXL promotes several processes, like cell survival by modulating NF-kB, by increasing anti-apoptotic expression such as survivin and B-cell lymphoma 2 (BCL-2) while conversely reducing the pro-apoptotic activity of Caspase 3 and BCL-2 antagonist of cell death (BAD) [70]. Given its various implications, AXL receptors have been studied in different forms of cancer. It has been noted the involvement of AXL in migration and invasion both in vitro and in vivo, as well as matrix metalloproteinase 9 (MMP9) is necessary for invading the surrounding cells [80].

In the literature, there are studies investigating the proto-oncogenic role of AXL focusing on the evaluation of these receptors to the epithelial-mesenchymal transition (EMT) process.

Fetal development and wound healing depend on the switchable process of EMT, in which cells follow a specific course to modify from an epithelial to a mesenchymal phenotype [81]. In healthy epithelial cells, cell-cell adhesion aids in preserving tissue integrity. A decrease in epithelial indicators like E-cadherin and a rise in mesenchymal markers like N-cadherin, Vimentin, α-catenin, and α-smooth muscle actin (α-SMA) are among the distinctive proteins implicated in EMT [82].

A study conducted on human breast cancer found that when SNAI2 (SLUG) and SNAI1 (SNAIL) are transfected into MCF10A cells, human breast cancer epithelial cells lose their epithelial-type shape and acquire mesenchymal-related characteristics [16], and this was linked to AXL increase. AXL expression is upregulated in human breast cancer epithelial cells, which results in the loss of epithelial-type shape and the acquisition of markers associated with mesenchymal tissue. In response to AXL deprivation, cells may exhibit an epithelial-type shape by decreasing the expression of transcription factors involved with EMT, promoting the production of E-cadherin and cell-cell adhesion as well as reducing the activity of TGF and Wingless-related integration site (WNT) signaling [16].

Vascular Endothelial growth factor (VEGF), fibroblast growth factor (FGF), and platelet-derived growth factor (PDGF) are all promoters of the angiogenic process. AXL is widely expressed in endothelial cells and vascular smooth muscle cells. It promotes the stabilization of aggregated platelets, survival of endothelial cells, and remodeling of endothelial barriers in wound healing and vessel impairment [83].

In the intricate pattern of tumors, there is a close correlation between the suppression of the immune system and their growth it is well known that most cancerous cells bypass the control of the immune system to proliferate [84]. Inhibiting the activity of natural killer cells (NK) and macrophages is essential for cells to continue with uncontrolled replication, and also for reducing the ability to recognize and eliminate metastases [84].

All three components of the TAM family are significant anti-inflammatory mediators that cascade block various pathways of activation of immune system cells such as macrophages and NK [85].

According to Rothlin and colleagues, the TAM receptors might act as a feedback mechanism to inhibit autoimmune reactions by suppressing the generation of cytokines and the TLR-dependent inflammatory response, seizing pro-inflammatory signals [85]. It has been established that AXL activation contributes to immune evasion by upregulating Twist and BCL-2, suppressing TLR inflammatory signaling, NK, and low production of pro-inflammatory cytokines [86].

Uncontrolled replication of cancerous cells often also leads to genetic changes involving new and different characteristics, it is not uncommon for tumors to develop resistance to therapy [87].

Furthermore, it has been observed that AXL is capable of causing innate or acquired resistance to certain forms of therapy (e.g., chemotherapy or immunotherapy) [88]. Documented cases of resistance to AXL-related drugs are in breast, colorectal and lung cancers [70]. Regardless of the location of tumor development, resistance to treatment involves multiple mediators such as extracellular signal-regulated kinase (ERK), PI3Kα, EGFR, and VEGF [70]. Understanding the structural and functional complexity of AXL, in all its aspects, thus becomes a keystone to exploit the various implications of this family of receptors.

## 4. Role of AXL Receptors in Glioblastoma

Increased expression of the receptor tyrosine kinase AXL is strongly associated with a poor prognosis in glioblastoma, according to a recent study by Sadahiro et al. [89]. They discovered that the mesenchymal (MES) glioblastoma subtype had increased expression of AXL. Additionally, they have investigated the theory that AXL signaling stimulates the formation of glioblastoma tumors by immune-suppressive extrinsic signals that alter the tumor microenvironment and internal mechanisms that control the survival and proliferation of glioma stem cells (GSC) [89].

The upregulation of AXL signaling is usually associated with glioblastoma development and progression [70]. Several studies advanced the carcinogenic role of the AXL receptor in the progression of glioblastoma. According to that, many clinical trials evaluating small molecule inhibitors of AXL in the treatment of recurrent glioblastoma have been registered on clinicaltrials.gov and are currently underway, as well, many combined treatments of anti-TAM therapy and other immunotherapeutic have been carried out [70].

Thus, a causative correlation may exist in which glioblastoma could be a direct result of overexpression of AXL receptors, and further studies are necessary to clarify this correlation.

On this basis, the present review aimed to analyze and summarize all the preclinical research that has been published from 2021 until now (written in English and performing a bibliographic search using Pubmed (MEDLINE) scientific database)) for a deeper comprehension of the extensive implications of AXL in the context of glioblastoma.

All the studies presented in this section are summarized in Table 1.

Understanding and better evaluating AXL could lead to the realization of drugs or chemical agents capable of counteracting glioblastoma progression.

It has been demonstrated that AXL and/or GAS6 overexpression were associated with a worse prognosis and more aggressive malignancy, as seen, for instance, in glioblastoma patients. Phospho-AXL is supposed to be widely activated in the glioblastoma [102]. Concerning this, the study conducted by Bielecka and colleagues identified the involvement of AXL and revealed its association with proteins that regulate the dynamics of actin [93]. They demonstrated that AXL activation triggers actin remodeling, leading to the formation of peripheral membrane ripples, circular dorsal ripples (CDRS) and micropinocytosis, contributing to the invasion and metastasis of cancer cells. Based on their demonstration, exploiting LN229 cells, glioblastoma may utilize AXL-driven micropinocytosis to some degree to ingest extracellular albumin and enable them to grow under glutamine conditions circumstances.

The activation of many actin-dependent processes by GAS6-AXL signaling has been shown to regulate the invasion of glioblastoma cells, and PI3K is a significant downstream effector of this signaling. In the past, PI3K was linked to the invasion of ovarian malignancies by AXL. These results imply that PI3K inhibitors, either alone or in conjunction with AXL inhibitors, may prevent malignant tumors, like glioblastoma, with highly active GAS6-AXL pathways from spreading metastatically [93].

The severity of glioblastoma requires innovative therapies capable of increasing survival and ensuring a high quality of life [103]. Immune checkpoint inhibitors have been fairly successful clinically such as lung cancer and melanoma, however, late-stage glioblastoma patients treated with this kind of immunotherapy did not exhibit any clinical improvement [99].

Chen and colleagues conducted a study in a mouse model of mesenchymal-like glioblastoma, they specifically focused on the role of cytotoxic T-lymphocyte–associated antigen 4 (αCTLA-4) and Anti-Programmed Death-1 (αPD-1) [99]. The outcomes demonstrated that the animals’ survival was extended by αCTLA-4 therapy, but not by αPD-1.

The therapeutic advantage of αCTLA-4 therapy was eliminated upon reduction of CD4^+^ T cells, indicating that this impact is reliant on these cells. Depletion of CD8^+^ T cells, on the other hand, did not affect the course of therapy.

Through the AXL/MER tyrosine kinase receptors, the researchers found that CD4^+^ T cells directly interacted with microglia, boosting Interferon-γ (IFNγ)-dependent activation and phagocytosis.

Specifically, it was discovered that these receptors are required for the tumor suppression that the CD4^+^ T cell-microglia circuit mediates. It’s interesting to note that the expression of major histocompatibility complex-II (MHC-II) molecules, which are involved in antigen presentation, was not necessary for the tumor cells to express CD4^+^ T cells’ anti-tumor function. Rather, it was discovered that the central nervous system’s microglia, a different kind of immune cell, and regular dendritic cells played important roles. MHC-II molecules, which are required for the CD4^+^ T cell response and tumor suppression, were expressed by both of these cell types [99]. The study of natural compounds and the development of drugs based on them remains a milestone of drug development.

Kim et al. described a study concerning the use of quercetin as a potential anticancer agent in glioblastoma. This bioactive flavonoid is present in different types of fruits, beverages, and herbal extracts [104]. It is also provides strong antioxidant and pro-apoptotic properties [105]. According to the findings, quercetin administration dramatically lowers the vitality of glioblastoma cell lines such as U87 and U373 while maintaining the survival of normal astrocytes, suggesting a possible preference for malignant cells. The research also looks into a yet unexplored impact of quercetin on the AXL/IL-6/STAT3 signaling pathway in the malignant microenvironment. This natural substance appears to induce apoptosis in glioblastoma cells without appreciably altering variables that promote cell proliferation, such as Akt or mitogen-activated protein kinases. It also decreases Interleukin-6 production, phosphorylation of Signal transducer and activation of transcription 3 (STAT3), and inhibits the expression of the AXL gene and protein in glioblastoma cells.

Further strengthening the suggested mode of action, the use of Short Harpin-RNA (shRNA) to inhibit AXL verifies its critical involvement in the apoptotic impact caused by quercetin [91].

Among natural compounds with anti-cancer activity, there is also corosolic acid, a natural substance extracted from Actinidia chinensis, which may have potential in the fight against glioblastoma. Different concentrations of this substance have been tested to evaluate the effect on cell viability, migration, and influence on F-actin, these effects are heavily conditioned by the GAS6/AXL and Janus kinase (JAK) axis. During the study, the ability of corosolic acid to stabilize the cell cytoskeleton was revealed, which turns out to be heavily modified during the glioblastoma and contributes to the aggravation and progression of this pathology [92]. Corosolic acid 20 μM notably reduced AXL and GAS6 expression in glioblastoma cell lines, also lowering the phosphorylated expression of JAK2, MEK and ERK. In addition, corosolic acid, blocked the typical abnormal cytoskeletal organization of glioblastoma by reducing the expression of F-actin [92]. Comprehensively, the inhibition of these factors resulted on suppression of glioblastoma cell migration and invasion [92].

Another study on glioblastoma cell cultures (using U118 and SF126 cell lines) investigated the relationship between AXL receptors and acquired resistance to chemotherapy or radiation therapy. As mentioned above, the standard therapy for glioblastoma consists of the combined use of TMZ and radiation. To assess the resistance to these forms of therapy, analysis of the C-terminal AXL (CT-AXL) was particularly important because it represents a marker of the post-transcriptional changes of AXL [97]. In order to replicate a chemo-resistant environment, Scherschinski and colleagues experimented with varying dosages of TMZ that enabled the cells to grow in a hypoxic environment. Their findings demonstrated that CT-AXL rises in response to TMZ doses given repeatedly and in hypoxic conditions; in this instance, resistance was caused by hypoxia-induced factor 1 (HIF1α), a well-researched radiation resistance trigger. The result of endogenous RTK-AXL overexpression was therapeutic resistance. Simultaneously, the combined impact of conventional therapy for glioblastoma and AXL-specific tyrosine kinase inhibitor (TKI) R428 (also known as Bemcentinib) was investigated. By selectively inhibiting the AXL component, it has been possible to prevent this receptor’s action, improve the efficacy of treatments, and avoid the resistance that radiation and TMZ have built up [97].

Additionally, Vo et al. examined U251 glioblastoma cell lines under hypoxic environments, which are thought to be strongly associated with AXL.

As the primary hypoxic component in the glioblastoma pattern, HIF1α activity was examined. It was also shown that a decrease in AXL activity is linked to a decrease in HIF1α levels and a decreased nuclear localization. Thus, HIF1 is linked to both the tumor’s enhanced growth and survival as well as its acquired resistance to treatments [101].

Innovative therapies for glioblastoma can also consider the use of oncolytic viruses, this type of treatment would be beneficial in those forms of cancer that develop resistance quickly or are difficult to approach surgically. Because oncolytic viruses have a strong tropism which enables them to selectively attack cancer cells, they are very helpful in the therapy of malignancies. Recent studies by Zwernik et al. have highlighted the oncolytic abilities of Zika virus (ZIKV) against glioblastoma. It has been determined that the receptor tyrosine kinase AXL serves ZIKV as an entry receptor in cancerous cells. These findings suggest that in AXL-expressing cell lines, ZIKV infection causes apoptotic cell death. The CRISPR knockout cell lines are resistant to infection and subsequent cell death because they lack a functioning AXL receptor.

The relationship between ZKIV, glioblastoma and AXL was also deepened by Pöhlking et al. [106]. According to their research, once ZIKV reaches glioblastoma cells, it multiplies intracellularly and causes widespread cytotoxicity [94]. The cell viability of LN-229 and LN-229 AXL^KO^ was measured using a WST-1 reagent for cell proliferation. ZIKV was added to both cell cultures, and it was discovered that AXL^KO^ cells did not exhibit any distinct cytopathic effects in comparison to LN-229 cells at any point in time or multiplicity of infection. This indicates that AXL appears to be necessary for ZIKV to enter the cells as well as for the infected cells to go through apoptosis, supporting the idea that ZIKV might be used as an oncolytic virus [94].

Chen and colleagues examined how AXL, Angiotensin-converting enzyme 2 (ACE2), dipeptidyl peptidase-4 (DPP4), Alanyl Aminopeptidase (ANPEP), Transmembrane serine protease 2 (TMPRSS2), and Glutamyl Aminopeptidase (ENPEP) could be carcinogenic as secondary pathways that the SARS-CoV-2 virus uses to its benefit as glioblastoma progresses. Both healthy individuals’ and COVID-19-infected patients’ brain samples from various regions were studied. When compared to other receptors, Asian people had greater levels of AXL and lower levels of ANPEP, although in healthy individuals, AXL had the greatest expression of all other receptors. Since glioblastoma is strongly associated with the immune system, AXL expression is favorably connected with large concentrations of immune system indicators such as monocytes and dendritic cells [95].

AXL signaling has been linked to several human cancer cases, and studies have demonstrated a negative correlation between overexpression as well as metastasis and prognosis. It has been shown that malignant human gliomas often overexpress AXL and/or GAS6, and that individuals with glioblastoma who have elevated levels of these proteins have a markedly shortened time to tumor growth.

Bielecka and colleagues tested selective AXL/TAM inhibitors (Bemcentinib, Gilteritinib and LDC1267) in glioblastoma cells. According to their research, LDC1267 has more potency and specificity as an AXL inhibitor when compared to the other two inhibitors, which have off-target effects on autophagy, endo-lysosomal, and cell proliferation.

To assess the blockage of this particular receptor type in the glioblastoma pattern, the phosphorylated forms of Protein Kinase B (AKT), ERK1/2, and AXL were examined. LDC1267 specifically showed IC50 values of 26, 25, and 48 nmol/L for the inhibition of ERK1/2, AKT, and AXL phosphorylation, respectively. When combined, these noteworthy findings show that whereas AXL is particularly needed for the GAS6-induced increase in cell growth, it is not necessary for the proliferation of LN229 cells under conventional culture conditions without its inclusion. Furthermore, glioblastoma LN229 cell proliferation is inhibited by Bemcentinib and Gilteritinib in a manner independent of AXL [98].

The tumor microenvironment has some characteristics such as a high degree of heterogenicity and a highly immunosuppressive context, for these reasons, increasingly innovative approaches are needed. Liu and colleagues investigated the effects of intracranial administration of (Z)-n-butylidenephthalide [(Z)-BP], released via Cerebraca Wafers (CWs), in combination with TMZ to target AXL and mTOR in the glioblastoma framework [96]. The treatment was developed in this study through the use of some cell lines, and established that the simultaneous inhibition of AXL, Mammalian target of rapamycin (mTOR) and EGFR were decisive in increasing IFNγ levels by decreasing the progression of cancer cells and stimulating the tumor environment to be more responsive to glioblastoma cells [96]. Synergistic treatment with TMZ inhibited downstream signals after mTOR, thereby reducing factors such as Programmed Cell Death Ligand 1 [96].

Well-proven glioblastoma treatments can be complemented by totally new and experimental approaches, often exploiting the properties of our immune system [107].

Kang and colleagues tried to identify possible target proteins using CAR-T cells.

CAR-T cells, like other forms of immunotherapy, could propose an optimal point of view in the treatment of all those tumors difficult to treat surgically or that develop quite quickly resistance-acquired to drugs, exactly like glioblastoma.

The target proteins chosen by CAR-T cells were preferred based on their high expression in glioblastoma and their high involvement in pathways that are often altered in cancer cells; the three investigated proteins are AXL, mesenchymal-epithelial transition factor (c-Met) and Folate receptor 1 (FORL1). Special chimeric antigen receptors CAR NK cell line (CAR-KHYG-1) was assembled to target specifically these proteins. The obtained results show that AXL and c-Met were overexpressed in most glioblastoma cell lines tested (11 out of 13), but not in neuroblastoma cells (0 out of 8).

FORL1, on the other hand, was overexpressed in 1 out of 16 cell lines. CAR-KHYG-1 cells have been able to selectively eliminate glioblastoma cells, thus demonstrating that AXL, c-Met and FORL1 can be very valuable therapeutic targets to counteract glioblastoma [90].

Another in vitro study examines the activity of glioblastoma cells in response to a radio-chemotherapy treatment, Lecoultre et al. noted that some receptors involved in phagocytosis (including AXL) are expressed more in glioblastoma cells following radio-chemotherapy [100].

A series of superficial receptors have been examined through RT-qPCR, which is considered useful to address phagocytic responses. It is assumed that a domino effect occurs wherein glioblastoma cells express more AXL when they come into contact with the extracellular fluid of nearby cells irradiated by radiotherapy [100]. Thus, expression of AXL during glioblastoma remains a key step for in-depth study [100].

**Figure 2:**
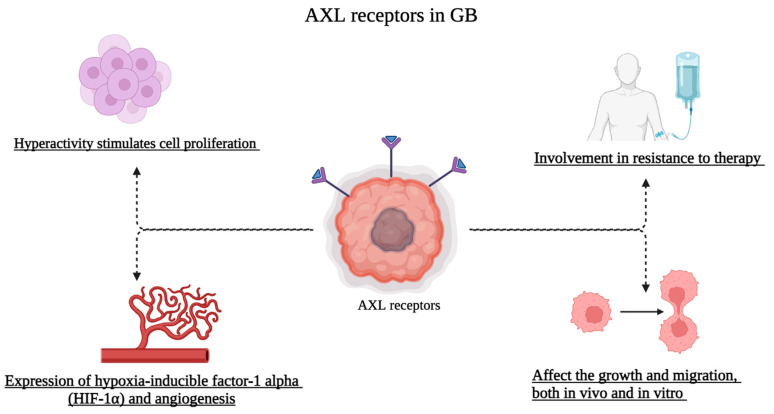
AXL functions in glioblastoma.

Therefore, based on what has been explained so far, the complex link between AXL and glioblastoma is well-understood and summarized in Figure 2. Deepening every facet of this relationship could allow us to develop innovative and better treatments that aim to improve outcomes for cancer patients.

## 5. Conclusions and Future Perspectives

The heterogeneous characteristics of glioblastoma represent a serious health problem for which it is urgent to identify innovative successful therapies. In this regard, AXL receptors are a great starting point to fully investigate alternative therapeutic routes to reduce pathological mechanisms of glioblastoma.

AXL would not only allow blocking of aberrant molecular signals upstream but also mitigation of the effects of immune-inflammatory pathways after their activation.

All the studies that explore the relationship of the AXL-glioblastoma axis should undoubtedly be supported and any approach, albeit unsuccessful, they could represent an interesting extra piece of knowledge in the complex biological network of glioblastoma.

Thus, future in-depth studies, both preclinical and clinical, fully exploiting AXL to counteract the progression of glioblastoma could represent a totally innovative type of therapy, since selective treatment for AXL could replace conventional therapies that unfortunately present several limitations and they are not always able to reduce the mortality rate of affected patients.

## Figures and Tables

**Table 1:** Studies involving the interplay between AXL and glioblastoma.

First Author and Year of Publication	Title	Therapeutic Target	Conclusion	Reference
Kang et al., 2021	Development of Antigen-specific Chimeric Antigen Receptor KHYG-1 Cells for Glioblastoma	CAR KHYG-1 cells can interact with c-Met, FOLR1, and AXL proteins	c-Met and AXL were over-expressed in several glioblastoma cell lines. CAR KHYG-1 cells can eradicate the positive cell of glioblastoma.	[90]
Kim et al., 2021	Quercetin Induces Apoptosis in Glioblastoma Cells by Suppressing Axl/IL-6/STAT3 Signaling Pathway	The role of quercetin on AXL/IL-6/STAT3 pathway is investigated.	They propose quercetin as a possible anticancer drug that might enhance cancer treatment.	[91]
Sun et al., 2021	Corosolic Acid Attenuates the Invasiveness of Glioblastoma Cells by Promoting CHIP-Mediated AXL Degradation and Inhibiting GAS6/AXL/JAK Axis	Authors have experimented with the use of corosolic acid to treat glioblastoma.	The data show that CA reduces the invasiveness of glioblastoma cells by interacting with AXL, GAS6 and JAK2/MEK/ERK cascade.	[92]
Zdzalik-Bielecka et al., 2021	The GAS6-AXL signaling pathway triggers actin remodeling that drives membrane ruffling, macropinocytosis, and cancer-cell invasion	The signaling pathway between AXL-GAS6 and how it affects the development of cancer cells.	Different actin-guided cytoskeletal rearrangements that the cell undergoes are caused by GAS6-AXL and contribute to the invasion of cancer cells.	[93]
Zwernik et al., 2021	AXL receptor is required for Zika virus strain MR-766 infection in human glioblastoma cell lines	AXL and ZIKV are highly involved and can be exploited to treat glioblastoma.	ZIKV entry into glioblastoma cells through the AXL receptor produces cytotoxicity.	[94]
Chen et al., 2022	Comprehensive Oncogenic Features of Coronavirus Receptors in Glioblastoma Multiforme	The authors study the connection between Coronavirus and glioblastoma, exploiting several receptors including AXL.	The work examines the connection between coronavirus receptors and glioblastoma for the first time and proposes the connection with ACE2, DPP4, ANPEP, AXL, TMPRSS2 and ENPEP	[95]
Liu et al., 2022	Targeting the Axl and mTOR Pathway Synergizes Immunotherapy and Chemotherapy to Butylidenephthalide in a Recurrent glioblastoma	The authors studied the connection between (Z)-BP delivery through CWs and TMZ in glioblastoma, focusing on AXL and mTOR.	Simultaneous treatment allows blocking of the progression of several cancer pathways.	[96]
Scherschinski et al., 2022	Regulation of the Receptor Tyrosine Kinase AXL in Response to Therapy and Its Role in Therapy Resistance in Glioblastoma	The role of AXL in the development of resistance-acquired therapy is explored.	RTK-AXL is required by the glioblastoma to develop drug resistance.	[97]
Zdzalik-Bielecka et al., 2022	Bemcentinib and Gilteritinib Inhibit Cell Growth and Impair the Endo-Lysosomal and Autophagy Systems in an AXL-Independent Manner	Bemcentinib and Gilteritinib are highly involved in autophagy by AXL.	The endo-lysosomal and autophagy systems were compromised by bemcentinib and gilteritinib in a way that was independent of AXL.	[98]
Chen et al., 2023	CTLA-4 blockade induces a microglia-Th1 cell partnership that stimulates microglia phagocytosis and anti-tumor function in glioblastoma	αCTLA-4 and αPD-1 are studied in a mouse model of glioblastoma.	In mesenchymal-like glioblastoma, αCTLA-4 inhibition activates CD4^+^ T cells and microglia via interferon-gamma.	[99]
Lecoultre et al., 2023	Radio-chemotherapy of glioblastoma cells promotes phagocytosis by macrophages in vitro	The work aims to evaluate the interplay of the actual treatment in glioblastoma also evaluating the AXL and other receptors	The effects of radio-chemotherapy on phagocytic activity may enhance pro-tumoral and anti-inflammatory TAM activities.	[100]
Vo et al., 2023	AXL is required for hypoxia-mediated hypoxia-inducible factor-1 alpha function in glioblastoma	AXL is involved in the release of (HIF-1α) in glioblastoma pattern.	HIF-1α and AXL co-expression was detected in human glioblastoma samples, but not in normal tissue.	[101]

## Data Availability

Not applicable.
